# The Impact of COVID-19 on the Orthopaedic Surgery Residency Match

**DOI:** 10.1055/s-0042-1755621

**Published:** 2022-09-19

**Authors:** Cees T. Whisonant, Shawhin R. K. Shahriari, Casey McDonald, Tyler Hough, Amanda C. Ederle, Gregory L. Borah

**Affiliations:** 1 Department of Surgery, Creighton University - Phoenix, Phoenix, Arizona; 2 Division of Plastic, Reconstructive, Hand and Burn Surgery, Department of Surgery, University of New Mexico, Albuquerque, New Mexico; 3 Department of Surgery, Creighton University - Phoenix, Phoenix, Arizona; 4 Department of Internal Medicine, Baptist Health, Louisville, Kentucky

**Keywords:** orthopaedic surgery, residency, the match, COVID-19 pandemic, graduate medical education

## Abstract

**Introduction** Matching into an orthopaedic surgery residency program presents a challenging accomplishment for applicants to achieve in any given year. Due to the profound changes to the application process caused by the coronavirus disease 2019 (COVID-19) pandemic it was theorized that there would be a change in the number of graduates matching close to their home medical school region, state, and program.

**Methods** Orthopaedic surgery residency program Web sites and social media accounts were accessed to elucidate current resident data, including graduates' medical school, and geographical location of their school. Chi-square analysis was performed to identify trends in current residents matching within their home program, state, and region associated with the 2021 orthopaedic match. These numbers were compared with previous year's successful applicants.

**Results** In 2021, a significant 4.4% ( *p* =0.02) increase in successful matches within applicants' home states occurred (33.4% vs. 37.8%) and home programs ( *p* <0.001) when compared with previous years (21.2% vs. 27.4%). However, in 2021, there was no significant change in home region matching ( *p* =0.56) with 60% of successful matches occurring in home regions. This was statistically consistent with what was observed in previous years (61.4%).

**Conclusion** The COVID-19 pandemic was associated with restrictions in travel and interview options resulting in a significant increase in the number of orthopaedic applicants who matched into their home program, or at programs in their home state compared with previous years. Although no statistically significant regional change occurred during the 2021 match, it remains the leading predictor of where successful applicants will match. With many unknowns related to the upcoming match cycles it is important for applicants and programs to have a general idea of recent trends and outcomes to best focus their efforts, especially if diversity and minority inclusion are considered in highly competitive specialties like orthopaedic surgery.

Orthopaedic surgery residency is a highly competitive surgical subspecialty and continues to attract the highest quality of applicants. When looking at the orthopaedic match in 2021, only 49.2% of applicants successfully matched. 1 Between 2008 and 2018, the number of applicants per position increased from 54.1 to 85.7. 2 With the sheer number of applications, it is an increasingly difficult task for program directors to determine who are the most suitable candidates for these limited and much sought-after residency positions. This has led to impersonal, statistical screening tools becoming common practice. Interestingly, the increased cost associated with an increased number of applications submitted has failed to deter students from applying widely. 2

The coronavirus disease 2019 (COVID-19) pandemic led to challenges for the medical school class of 2021, as they faced pandemic-related constraints on limiting interview travel and on prohibitions on away rotations as well as in-person interviews. A strong network of in-person connections has previously been demonstrated as essential for successful residency matching, especially in individuals reapplying to orthopaedic surgery. 3 The restrictive changes including virtual interviews and limitations on away rotations due to the global pandemic made making a meaningful connection with mentors outside of one's medical school significantly more difficult. Not only did it decrease program's access to applicants to evaluate them, it decreased the ability of applicants to get a feel for various programs.

The purpose of this study was to determine if the COVID-19 pandemic restrictions affected the 2021 match in orthopaedic surgery residency. Our goal was to determine what changes occurred in terms of where successful students matched, thereby providing insight into the current state of the match process. Given that students could not perform away rotations or attend in-person events, such as conferences and interviews, we hypothesized that there would be an expected increase in medical students matching in familiar programs (i.e., their home medical school programs, within the same state, or within the same region). The Coalition for Physician Accountability recently released updated recommendations for programs to continue to host virtual interviews with applicants for the class of 2022. With this in mind, this information will be important for applicants and programs in the coming Match cycles to evaluate how these similar changes previously affected the residency application process, especially in 2022.

## Methods

A list of accredited orthopaedic surgery residency programs and positions offered between 2017 and 2021 was collected from the National Resident Matching Program Web site. 4 Matched applicants and their medical school were obtained from residency program's Web sites and social media accounts including Instagram and Twitter and tabulated. Regionality (South, West, Northeast, and Midwest) was assigned to every orthopaedic surgery residency program and to each medical school based upon the U.S. Census designation. Medical school and residency program region and state for successfully matched applicants were compared, in addition to reviewing the number of applicants who matched at their home institution. Chi-square analysis was used to determine whether any significant changes occurred in terms of residents matching within their home program, state, or region, first looking at the data annually, then comparing the pre-COVID match (2017–2020) to the COVID match (2021).

## Results

Data from a total of 3,740 successfully matched orthopaedic surgery residents was collected and analyzed for the years 2017 to 2021, which accounts for 94.6% of residency positions. Data, including total number of matched applicants per year, home program, home state, and home region matches are summarized in Table 1 . The composition of males and females in accepted applicants was not found to be affected in 2021 compared with previous years. On average, 80% of matched applicants were male and this number did not change significantly during the study period.

**Table 1:** Year effect on home program/state/region matching

Year	*N*	Home program		Home state		Home region		
		*n*	%	*n*	%	*n*	%
2017	707	156	22.1	249	35.2	457	64.6
2018	730	140	19.2	223	30.5	439	60.1
2019	737	161	21.8	251	34.1	443	60.1
2020	809	174	21.5	273	33.7	491	60.7
2021	747	205	27.4	282	37.8	448	60.0
2017–2021		*p* -value=0.0030 a		*p* -value=0.0602		*p* -value=0.2474		
2017–2020		*p* -value=0.5341		*p* -value=0.2880		*p* -value=0.1659		
2017–2020 vs. 2021		*p* -value<0.001 a		*p* -value=0.0211 a		*p* -value=0.5579		

a Indicates statistical significance.

### COVID-19 Pandemic Effect on Home Program Matching

As seen in Table 1 , a significant increase in the number of applicants matching at their home programs, the program affiliated with their medical school, was found between 2017 and 2021. When the data was further analyzed to exclude 2021, however, no significant change was determined. When the results from successful matches between 2017 and 2020 were compared against matched applicants from 2021, a significant increase of 6.3% of orthopaedic surgery residents successfully matched at their home programs.

### COVID-19 Pandemic Effect on Home State Matching

**Fig. 1:**
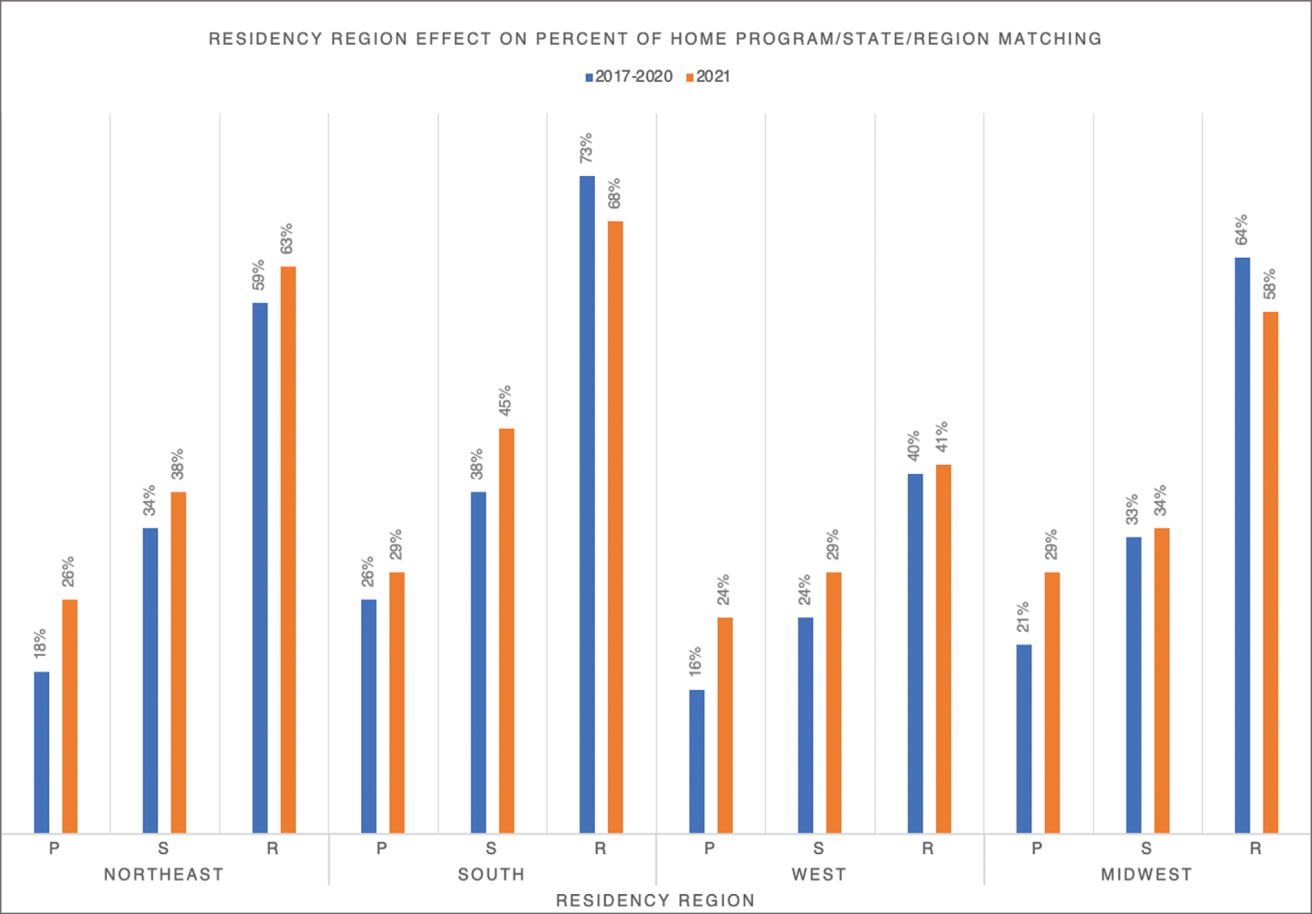
The effect of residency region on the percent of home program, state, and regional matching between 2017 and 2020 versus 2021. P, Program; S, State; R, Region.

Also seen in Table 1 , a significant increase of 4.5% occurred in applicants who matched within the same state as their medical school when comparing 2021 to previous years. The largest increase in home state matching was found in programs in the South while the region that changed the least was in the Midwest, seen in Fig. 1 .

### COVID-19 Pandemic Effect on Home Region Matching

The Northeast region showed a 4% increase in the number of applicants matching within the same region ( Fig. 1 ), while the South and Midwest showed a decrease in the number of home region matching by 5 and 6%, respectively. No significant change was identified in terms of same region matching when comparing 2021 to previous years, as demonstrated in Table 1 .

## Discussion

Between 2017 and 2021, there was an increase from 150 to 188 accredited orthopaedic surgery residency programs. There was also an increase of 134 available residency positions during this period, from 732 to 866 positions. The South was found to have the most residency programs (51–57) and residency positions (225–239) per year. Overall, the South and Midwest had more successful applicants than residency positions available in their respective regions, while the Northeast and West had more residency positions available than successful applicants ( Table 2 ). On average, 71.7% of residents at programs in the South graduated from medical schools in the South; this is the most of any region.

**Table 2:** Residency positions versus applicants by region

	Medical school graduates	Residency positions	
Northeast	952	1,081
South	1,369	1,150
West	391	554
Midwest	972	955

The number of applications relative to available positions have increased over time, as have the competitive qualifications of applicants as demonstrated by a steady increase in scores on the United States Medical Licensing Examination (USMLE) Step 1 score and research experience. 5 Previous studies have demonstrated that location is a major factor in the match as students often apply to and rank institutions based on their own hometown, undergraduate institution, and medical school. 6 7 Our data similarly demonstrates a positive association between the geography of graduates' medical schools and residency program ( Table 1 ).

### Home Program

In orthopaedic surgery, 21% of applicants matched at their home program (HP), that is, the residency program affiliated with their medical school, from 2017 to 2020, and in 2021 this increased by 6.4% to 27.4% ( Table 1 ). This demonstrated almost a 30% increase in the overall number of applicants matching at their home institutions in the study period from 156 HP matches in 2017 to 205 HP matches in 2021. Similarly, there was an 8.4% increase in home program matches observed when looking at integrated plastic surgery residency, another competitive surgical subspecialty. 8 These trends are consistent with what many have hypothesized with the changes that occurred due to COVID-19.

Interestingly, there was also an increase in home program matching that was noted across all regions with the largest increase being in the Northeast and the West. This may be due to factors such as not being able to perform away rotations, along with home programs having increased familiarity with their own students. Additionally, students may feel more comfortable ranking their home program. As the Match is not a one-sided decision, applicants may also have desired to stay closer to their home medical school during these uncertain times and due to travel restrictions, which could have led to increased matches not only within an applicant's home program, but also their home state.

### Home State

A significant increase of applicants matching within their home state occurred in 2021, especially in the South where nearly half of all successfully matched applicants graduated from southern medical schools ( Fig. 1 ). From 2017 to 2020, an average of 33% of applicants matched within their home state. In 2021, this percentage increased to 38%. This was once again comparable to what was noted in the integrated plastic surgery match in 2021. 8

As seen in Table 3 , the states most likely to match instate graduates (by number of positions) are New York, California, Pennsylvania, Texas, and Michigan. Texas programs matched the highest percentage of instate applicants between 2016 and 2020 ( Table 3 ). This remained true in 2021, with over 75% of matched applicants in Texas having graduated from a Texas medical school. It is unclear why so many applicants from these states remain in their home state, but it could likely be explained by the large number of medical schools and thus applicants in those states. Programs and applicants likely relied on their word of mouth and familiarity and experience from graduates from these medical school programs, resulting in matching students from neighboring institutions.

**Table 3:** States most likely to match same state graduates

State	2016–2020		2021	
	Positions	Home state (%)	Positions	Home state (%)
New York	381	146 (38.3)	95	46 (48.4)
California	262	74 (28.2)	56	17 (30.4)
Pennsylvania	210	76 (36.2)	50	18 (36.0)
Texas	199	122 (61.3)	53	40 (75.5)
Michigan	158	54 (34.2)	48	16 (33.3)

### Home Region

Residency programs in the South were found to match same region applicants the most often between 2016 and 2020 ( Fig. 1 ). Although this remained true in 2021, there was a 1.4% overall decrease in applicants matching within their own region with the largest decrease in regional matches being noted from the Midwest and South. It is unclear as to why this may have happened, especially since both home program and home state match rates increased.

Limitations of this study are largely due to the way in which data was collected. While the overall response rate was 94.6%, this data was collected from residency Web sites and social media accounts. Possible confounders include where students completed any away rotations or research years, factors that could have facilitated connections with the programs they matched with. Our data also does not look at other aspects of matched residents' applications, such as letters of recommendation, clinical evaluations, Deans' letters, or USMLE scores. It will be interesting to see how these trends change over time as the USMLE Step 1 moves to a pass/fail format. These are essential parts of the application process and analyzing these in future studies will be interesting and valuable. It was not investigated how traditionally underestimated minority applicants or applicants without a home program were affected by the pandemic; however, these would be areas of interest for future studies.

## Conclusion

Analyzing trends in the Match process is helpful for both medical student applicants and residency program directors. This information helps students try to determine where they are most likely to match and where they can help focus their application efforts. These factors should also be considered when program directors advise their students on possible application sites. Many applicants successfully match into residency programs located in the same region as their medical school, and this did not change significantly during the COVID-19 pandemic. Conversely, there was a significant increase in the number of graduates matching into orthopaedic residency programs within the same state and at their home programs this past year. It is unclear the long-term ramifications the Match will experience due to COVID-19 and whether restrictions on away rotations will continue, but it is likely that virtual interviews will become a more utilized tool in future match cycles. As residency programs attempt to increase the diversity within their programs, it may be hindered by the recent trends in applicants remaining close to home. With the continuation of virtual interviews and the restriction on away rotations for applicants, the 2022 Match cycle will likely mirror that of the 2021 cycle. As such, applicants and programs would benefit to know how a similar situation previously affected the residency application and match process. Knowledge of this may assist future applicants on places they are most likely to match and may help to narrow not only where they apply for away rotations but also where they should focus their efforts and money when applying to residency.
